# 4-Vinylguaiacol in Citri Reticulatae ‘Chachiensis’ Pericarpium Volatile Oil: A Microbial-Mediated Aging Marker Enhances Glucose Metabolism

**DOI:** 10.3390/foods14203489

**Published:** 2025-10-14

**Authors:** Hao Zheng, Zhi-Cheng Su, Shu-Ting Huang, Dong-Li Li, Zhao-Dong Yuan, Ju-Cai Xu, Ri-Hui Wu, Li-Gen Lin, Li-She Gan

**Affiliations:** 1 School of Pharmaceutical Sciences, Zhejiang Chinese Medical University, Hangzhou 311402, China; zhengh_ao@163.com; 2 State Key Laboratory of Quality Research in Chinese Medicine, Institute of Chinese Medical Sciences, University of Macau, Macao 999078, China; ligenl@um.edu.mo; 3 School of Pharmacy and Food Engineering, International Healthcare Innovation Institute, Wuyi University, Jiangmen 529020, China; wyuchemszc@126.com (Z.-C.S.); huangst0808@163.com (S.-T.H.); wyuchemldl@126.com (D.-L.L.); wyuchemyzd@126.com (Z.-D.Y.); xujucai.happy@163.com (J.-C.X.); wyuchemwrh@126.com (R.-H.W.)

**Keywords:** Citri Reticulatae ‘Chachiensis’ Pericarpium, aging process, 4-vinylguaiacol, glucose uptake, metabolomics, microbial biotransformation

## Abstract

Influenced by various physical, chemical, and microbial factors, the aging process of Citri Reticulatae ‘Chachiensis’ Pericarpium (CRCP) poses a complex scientific challenge. Drawing inspiration from the perspective of traditional Chinese medicine, volatile oils were extracted from CRCP aged 1, 3, 5, and 7 years by steam distillation and subsequently analyzed by GC-MS. The results revealed that the relative percentage of 4-vinylguaiacol (4-VG) increased progressively with aging. Nineteen volatile oil components were further assessed for their glucose metabolism-enhancing activities, with 4-VG emerging as a key active compound. Notably, 4-VG remarkably enhanced insulin-stimulated glucose uptake in C2C12 myotubes. Moreover, 4-VG demonstrated potent antihyperglycemic effects by upregulating IRS-1/Akt/GSK-3β phosphorylation in the insulin signaling pathway on a high-fat diet and STZ-induced diabetic mouse model. In addition, the metabolic pathway of 4-VG, from ferulic acid and then to vanillin and guaiacol, was verified via HPLC-UV, metabolomics, and microbiome analyses, which confirmed the microbial conversion of 4-VG within CRCP. The metabolic pathway was ultimately validated by isolating and identifying Priestia aryabhattai, Bacillus velezensis, and Aspergillus fumigatus from CRCP, with further in vitro culture and biotransformation experiments confirming its functionality and efficiency. These findings provide new insights and experimental evidence that deepen our understanding of the aging process of CRCP.

## 1. Introduction

The dried and aged pericarp of *Citrus reticulata* Blanco, commonly known as Citri Reticulatae Pericarpium (CRP), or ChenPi in Chinese, was first documented in the Tang Dynasty classic, *Dietetic Material Medica* (621-713 A.D.), in which its therapeutic effects on gastrointestinal disorders were highlighted. Today, CRP is extensively utilized in both traditional Chinese medicine and the food industries across China, Japan, and other Southeast Asian countries [1]. A particular variant of CRP, known as Citri Reticulatae ‘Chachiensis’ Pericarpium (CRCP), or GuangChenPi in Chinese, holds a special status due to its rarity and exceptional health benefits [2]. CRCP is derived from the ‘Chachiensis’ variety of *C. reticulata*, mainly cultivated and aged in Xinhui District, Jiangmen City, Guangdong Province. Recognized for its unique qualities [3], CRCP was officially acknowledged in the 2020 edition of the Chinese Pharmacopoeia, fostering a specialized cultivation and trade industry valued at over 30 billion USD annually.

CRP and CRCP have been extensively investigated, leading to the identification of over 200 compounds [4], including polymethoxyflavones, volatile oils, polysaccharides, phenolic acids, and alkaloids. The polymethoxyflavones have demonstrated promising antiviral activity [5] and strong protective effects against oxidative damage in HepG2 cells [6]. Our previous studies found that CRCP volatile oil exhibits anti-mosquito activity in animal models [7], while the polysaccharides presented anti-obesity potential by modulating gut microbiota [8]. These findings highlight the diverse therapeutic potential of various components from CRCP.

CRCP must undergo an aging process of over three years before use [9]. In traditional Chinese medicine, this aging process is believed to reduce the pungent and hot odor of the fresh pericarps, mellowing their properties, in which volatile oils may play a key role in the remarkable transformation of odors and flavors. In Xinhui district, local people used to place edible oil in the concave area of CRCP and heat it using an oil lamp or candle. This process allowed for the extraction of volatile oils, which were then applied in massage treatments for children’s abdominal pain. Therefore, the efficacy of CRCP volatile oils may be closely linked to their traditional use in improving body metabolism.

Throughout its aging process, CRCP undergoes both physical and chemical transformations, as well as microbial fermentation, all of which contribute to its complexity [10]. Physically, the pericarps dry and volatile components evaporate [11,12]. Chemically, oxidation and decomposition occur with exposure to air and sunlight. Microbial fermentation leads to alterations in both the microbial community and metabolite profiles [13,14]. Together, these changes form a multifaceted aging process that presents a significant challenge to researchers.

Recent studies have advanced our understanding of CRCP’s aging process. CRCP aged for over 5 years showed higher content of polymethoxy flavonoids (PMFs) and exhibit greater bioactivity in spleen-deficient mice [15]. Variations in free PMFs during storage have been linked to differing anti-cancer activities [16]. Additionally, molecular weights of polysaccharides decrease during aging, resulting in enhanced immunomodulatory effects [17]. Furthermore, a proposed biosynthetic pathway suggests that the characteristic compound methyl N-methylanthranilate undergoes methylation, oxidation, and subsequent coupling transformations into colored pigments such as N,N’-dimethylindigo and N, N′-dimethylindirubin, which explains the characteristic browning of aged CRCP [18]. Short-term steaming for 60 s has been shown to reduce irritation in fresh pericarps and increase flavonoid content [19], while non-volatile compounds vary depending on drying conditions [20]. The volatile oil composition throughout the aging process is closely associated with coexisting microbial populations and communities, which are influenced by environmental factors [2,21]. Key aroma compounds, such as α-phellandrene and nonanal, may contribute to flavor changes during aging [22]. Characteristic microorganic flora is considered one of the crucial factors contributing to the authenticity of CRP in a specific region and plays a significant role during CRP storage. Certain bacteria and fungi, such as *Aspergillus niger* and *Penicillium simplicissimum*, may facilitate the transformation of flavonoids and accelerate the aging process of CRCP [11,23]. High-throughput sequencing and comparative metabolomics studies have shown that *Bacillus thuringiensis* and *Xeromyces bisporus* are strongly correlated with the key active metabolites [11].

In this study, volatile oils from CRCP aged 1, 3, 5, and 7 years were extracted via steam distillation and analyzed using GC-MS. Nineteen volatile oil components were selected for further investigation of their glucose metabolism-enhancing efficacy. Among them, 4-vinylguaiacol (4-VG), an oily flavor compound notable for its unique spicy and clove-like aroma found in various beverages and foods, demonstrated a trend of increasing relative content with the aging years and exhibited remarkable glucose uptake-enhancing activity. This promising result prompted its further assessment in a high-fat diet and STZ-induced diabetic mouse model. Furthermore, a metabolic pathway involving the microbial transformation of ferulic acid to 4-VG and then to vanillin and guaiacol in CRCP was verified through HPLC-UV, metabolomics, and microbiome analyses. Isolation and identification of microorganisms from CRCP, alongside in vitro bio-transformation experiments, further confirmed the existence of this metabolic pathway.

## 2. Materials and Methods

### 2.1. Reagents and Materials

CRCP samples were obtained from Guangdong Jiangmen Pipibao Chenpi Co., Ltd. (Xinhui District, Jiangmen, China) and authenticated by one of the authors, professor Dong-Li Li. Nineteen components of CRCP volatile oil, including α-pinene (CAS: 80-56-8, AR), β-pinene (CAS: 127-91-3, AR), β-myrcene (CAS: 123-35-3, AR), D-limonene (CAS: 58555-74-1, AR), γ-terpinene (CAS: 99-85-4, AR), terpinolene (CAS: 586-62-9‌, AR), terpinen-4-ol (CAS: 562-74-3, AR), linalool (CAS: 78-70-6, AR), α-terpineol (CAS: 10482-56-1, AR), decanal (CAS: 112-31-2, AR), carveol (CAS: 99-48-9, AR), perillaldehyde (CAS: 18031-40-8, AR), thymol (CAS: 89-83-8, AR), p-thymol (CAS: 3228-02-2, AR), carvacrol (CAS: 499-75-2, AR), 4-VG (CAS: 7786-61-0, AR), methyl 2-(methylamino)benzoate (CAS: 85-91-6, AR), β-caryophyllene (CAS: 87-44-5, AR), and α-sinensal (CAS: 17909-77-2, AR), were purchased from Jiangxi Hailin Perfume Co., Ltd. (JiAn, Jiangxi, China), with over 98% purity.

The compounds were dissolved in dimethyl sulfoxide (DMSO, SigmaAldrich, St. Louis, MO, USA) to make a 10 mM stock solution and stored at −20 °C. The experimental concentration of DMSO was below 0.1%. Dulbecco’s modified Eagle’s medium (DMEM), fetal bovine serum (FBS), horse serum (HS), Penicillin-Streptomycin (P/S, 10,000 units/mL of penicillin and 10,000 μg/mL of streptomycin) and phosphate-buffered saline (PBS) were purchased from Gibco (Carlsbad, CA, USA). 2-(N-(7-nitrobenz-2-oxa-1,3-diazol-4-yl) amino)-2-deoxyglucose (2-NBDG) and 3-(4,5-dimethyl-2-thiazolyl)-2,5-diphenyltetrazolium bromide (MTT) were purchased from Sigma-Aldrich (St. Louis, MO, USA). Primary antibodies against IRS-1, p-IRS-1, GSK3β, p-GSK3β, p-Akt, Akt and GAPDH, as well as secondary antibodies, were obtained from Cell Signaling Technology (Beverly, MD, USA).

### 2.2. Steam Distillation Extraction and GC-MS Analysis of CRCP Volatile Oil

The CRCP was first ground and sieved (60-mesh), and then 20 g of the powder were placed in a three-neck flask equipped with a volatile oil extractor. Ten times of distilled water was added, and the mixture underwent reflux extraction twice at 95–100 °C, each for two hours. Subsequently, the extracted volatile oils were combined and dried by an appropriate amount of anhydrous sodium sulfate. The yields of the extracted volatile oils were approximately 0.3%. Three biological replicates were processed and pooled.

For the GC-MS analysis of the volatile oil, a DB-5 (30 m × 250 μm × 0.25 μm) elastic quartz capillary column in a Agilgent 8890 GC System (5977B GC/MSD, Santa Clara, CA, USA) was used for chromatography. An aliquot of 50 mg of the volatile oil was dissolved in 0.5 mL of methanol in a 2 mL centrifuge tube (Axygen, Union, CA, USA). The mixture was sonicated and then filtered. A volume of 1 μL of the resulting solution was injected for analysis, with each sample injected in triplicate. The following chromatographic conditions were applied: The initial oven temperature was set at 70 °C and held for 2 min. The temperature was then increased at a rate of 5 °C/min to 160 °C, followed by a further increase at a rate of 20 °C/min to 240 °C, where it was held for 4 min. The total run time for the analysis was 26 min. The injection temperature was set at 230 °C, and the flow rate was maintained at 1.3 mL/min using high-purity helium as the carrier gas. The split ratio used was 10:1.

### 2.3. Insulin-Stimulated Glucose Uptake Assay

Mouse C2C12 myoblasts (RRID: CVCL_0188) were obtained from ATCC and maintained in DMEM with 10% FBS and 1% P/S. To initiate differentiation, the cells were grown to 100% confluence and incubated with DMEM containing 2% heat-inactivated HS and 1% P/S for 4 days. Media were refreshed every day to provide sufficient nutrition. The fully differentiated myotubes were used for the following experiments.

Myotubes were incubated with compounds at a final concentration of 5 μM that was set based on our previous assays [24,25]. Then, cells were washed with Krebs–Ringer phosphate (KRP) buffer (20 mM of HEPES, 137 mM of NaCl, 4.7 mM of KCl, 1.2 mM of MgSO_4_, 1.2 mM of KH_2_PO_4_, 2.5 mM of CaCl_2_, and 2 mM of pyruvate; pH 7.4) and incubated in KRP buffer with 0.2% BSA for 2 h. To stimulate glucose uptake, cells were incubated with KRP buffer containing 0.1 μM of insulin for another 30 min. After washing with KRP buffer once, cells were incubated in KRP containing 100 μM of 2-NBDG (Sigma-Aldrich) for 30 min. The intracellular amount of 2-NBDG was measured at an excitation wavelength of 475 nm and an emission wavelength of 550 nm. 5-Aminoimidazole-4-carboxamide ribon ucleotide (AICAR, 20 μM), an AMPK activator, was used as the positive control.

For Western blotting experiments, protein concentration of each sample was quantified by BCA protein assay kit. The same amount of protein (30–50 μg) was separated by SDS-PAGE, transferred to PVDF membranes (Bio-Rad, Hercules, CA, USA), blocked with 5% nonfat milk in TBST buffer (100 mM of NaCl, 10 mM of Tris–HCl, pH 7.5, and 0.1% Tween 20) for 2 h at room temperature, and incubated with specific primary antibodies overnight at 4 °C. After washing with TBST three times, an HRP-conjugated secondary antibody was added and incubated for another 2 h at room temperature. The immunoblotting signals were developed using a SuperSignal West Femto Maximum Sensitivity Substrate kit (Thermo, Rockford, IL, USA). Then, specific protein bands were visualized using the ChemiDoc MP Imaging System (Bio-Rad, Herakles CA, USA) and quantitated using Image Lab 5.1 (Bio-Rad).

### 2.4. HFD Plus STZ-Induced Diabetic Mice Model

All animal care and experimental procedures were approved by the Animal Ethical and Welfare Committee of International Healthcare and Innovation Institute (Jiangmen, China) and performed in accordance with the guidelines and regulations of this institution. Male C57BL/6J mice (4 weeks old) were purchased from Zhuhai Bestest Bio-Tch Co., Ltd., (Zhuhai, China). The mice were housed at 22 ± 1 °C with 12 h light–dark cycles and fed with a regular chow diet (RD; Medicience Ltd., Yangzhou, Jiangsu, China) and water ad libitum under standard conditions (specific-pathogen-free) with air filtration.

Male C57 mice (four-week-old) with an initial body weight of 8–10 g was fed with a RD for three days and randomly divided into two groups. NC group was fed with a RD (calorie, 3.5 kcal/g), and HFD group was fed with a 45% HFD (calorie, 16.8 kcal/g, Xiao Shu You Tai Biotechnology Co., Beijing, China). Diabetic mice were induced by STZ after 5 weeks of HFD feeding. After fasting for 12 h, NC group was intraperitoneally injected with citric acid-sodium citrate buffer solution (0.1 mM, pH4.5, 10 mL/kg), and HFD group was intraperitoneally injected with STZ dissolved in citric acid-sodium citrate buffer solution (40 mg/kg, 10 mL/kg) for 7 days. Mice were maintained for 10 days. Mice with fasting blood glucose above 11.1 mM on 10th day, above 13 mM on 12th day and above 13 mM on 14th day after STZ injection were considered to be diabetic mice for the following experiments. HFD/STZ mice were randomly divided into 3 groups. Mice in 4-VG group and acarbose (ACA) group were gavaged with 4-VG (50 mg/kg) and ACA (50 mg/kg) once a day for 4 weeks, respectively. Body weight and energy intake were monitored weekly. 24 h after the last gavage, blood samples were collected from orbital sinus after deep anaesthesia with CO_2_ suffocation. Mice were euthanatized by CO_2_ inhalation and gastrocnemius was dissected.

Levels of total cholesterol (TC), triglyceride (TG), and low-density lipoprotein-cholesterol (HDL-C) in serum were analyzed by commercial kits (Nanjing Jiancheng, Nanjing, Jiangsu, China) according to manufacturer’s instruction. Glucose tolerance tests (GTT) were performed after treatment for 25 days as previous methods with slight modifications [26,27,28]. Briefly, after 12 h fasting, the tail blood glucose was measured by Roche blood glucose meter and test strips. Next, mice were administered glucose via gavage at a dosage of 2.0 g·/kg. The tail blood glucose was measured at 15, 30, 60, 90, and 120 min after gavage.

### 2.5. Determination of the Contents of Ferulic Acid, 4-VG, Vanillin, and Guaiacol in 1, 3, 5, 7-Year CRCP by HPLC-UV

1.000 g of the powdered 5-year CRCP sample was transferred into a 10 mL volumetric flask. The flask was then filled with a mixed solvent comprising methanol and ethyl acetate in a 5:1 ratio and was sonicated for 20 min to ensure thorough extraction. The volume was adjusted back to the calibration mark with the same mixed solvent and the solution was then filtered through a 0.22 μm organic membrane filter for subsequent analysis. The extractions were performed three times. Multiple biological replicates were processed and pooled.

0.0040 g Vanillin, 0.0040 g ferulic acid, 0.1100 g guaiacol, and 0.0120 g 4-VG were precisely weighed and, respectively, transferred to 10 mL volumetric flasks. The volumes were brought up to the mark with methanol, and the solutions were then filtered for analysis.

High-Performance Liquid Chromatography analyses were performed using a UHPLC ACOUITY Arc system (Waters, Milford, MA, USA) equipped with a 2998 UV/visible photodiode array detector. Column: ACQUITY UPLC BEH C18 (150 × 4.6 mm, 5 μm); Column Temperature: 35 °C; Mobile phase: 0.1% phosphoric acid in water (A)-acetonitrile (B); Gradient Elution: 0–6 min, 15% B; 6–10 min, 15–35% B; 10–12 min, 35–50% B; 12–20 min, 50% B; 20–25 min, 50–100% B; 25–30 min, 100% B; Flow rate: 1.0 mL/min; UV-Detector detection wavelength: 230 nm; Injection volume: 10 μL for the CRCP samples, and for the standard compounds (ferulic acid, 4-VG, vanillin, and guaiacol), 0.12, 0.22, 0.97, 3.78, 7.65, and 10.52 μL, respectively.

### 2.6. DNA Extraction, Illumina Miseq Sequencing, and Microbiomics Data

Microorganisms (including endophytes) in CRCP samples were collected by grinding 5 g of CRCPs with liquid nitrogen and adding to a 500 mL flask containing 100 mL of sterile saline (0.9% NaCl) at 4 °C with shaking at 220 rpm for 4 h. After shaking, the CRCP pellets were removed with four layers of gauze and centrifuge at 8000 rpm for 10 min to collect microbial spheroids. The total genomic DNA of the sample was extracted using a TGuide S96 kit (DP812, Tiangen Biotech Co., Ltd., Beijing, China). Amplify the V3-V4 region of the bacterial 16S rRNA gene with forward primer 338F (5′-ACTCCTACGGGAGGCAGCA-3′) and reverse primer 806R (5′-GGACTACCGGGTATCTAAT-3′). Amplify the fungal ITS1 region with forward primer ITS1 (5′-CTTGGTCATTTAGAGGAAGTAA-3′) and reverse primer ITS2 (5′ -GCTGCGTTCTTCATCGATGC-3′). The PCR reaction comprised the following components: genomic DNA (5 to 50 ng), forward primer (0.3 μL), reverse primer (0.3 μL), KOD FX Neo Buffer (5 μL), dNTP mix (200 μM each) (2 μL), KOD FX Neo (1 U/μL) (0.2 μL), and ddH_2_O added to a final volume of 10 μL. The polymerization reaction conditions were: 95 °C for 5 min (initial denaturation), 95 °C for 30 s (denaturation), 50 °C for 30 s (annealing), 72 °C for 40 s (elongation). The denaturing-annealing-stretching step was repeated for 25 cycles, followed by 72 °C stretching for 7 min to end the scale-up. Finally, total PCR products were quantified by Quant-iT™ dsDNA(Thermo Fisher Scientific, Waltham, MA, USA) HS Reagent and put together. High-throughput sequencing analysis for bacterial rRNA genes was operated on the purified, pooled sample by using the Illumina Hiseq 2500 platform (2 × 250 paired ends) at Biomarker Technologies Corporation, Beijing, China.

Raw fastq files were demultiplexed, quality-filtered by Trimmomatic and merged by FLASH Operational taxonomic units (OTUs) were clustered with 97% similarity cutoff using UPARSE. The alpha diversity indices, rarefaction curves, and the number of unique OTUs in each sample were evaluated using the Mothur V1.45.3software. For beta diversity analysis, Principal component analyses (PCA) were performed using QIIME2 software [29]. Linear discriminant analysis Effect Size (LEfSe) analysis (https://huttenhower.sph.harvard.edu/galaxy/, accessed on 5 October 2025) was used for searching characteristic fungal (ITS) and bacterial (16S rDNA) taxonomical biomarkers between four aging year groups of the CRCP. The thresholds of the linear discriminant analysis (LDA) values were set as 4 for 16S rDNA data and ITS data, respectively. Spearman rank correlations were calculated using the psych package in R version 2.2.5.

### 2.7. Isolation and Identification of Microorganisms in CRCP

Under sterile conditions in a laminar flow hood, a 10 g sample of CRCP was finely chopped using sterile scissors into a sterile bag. 1.0 g of chopped CRCP was rinsed twice with sterile water and subsequently placed in a 50 mL centrifuge tube. 5 mL of sterile saline solution was added, and the tube was shaken at 200 rpm at 28 °C for 1 h to yield a microbial suspension. 400 μL of the suspension was applied onto solid media plates of LB, YPD, and PDA. The LB plates are incubated at 37 °C, while the YPD and PDA plates are maintained at 30 °C. After 24 h, colonies exhibiting varied morphologies were selected from these plates, and isolation was achieved by repeated streaking to secure pure cultures. A single colony from each purified culture was then transferred to the corresponding liquid medium and incubated at either 37 °C or 30 °C at 200 rpm to cultivate single colony suspensions.

Strain identification involves several steps starting with DNA extraction. Cultures grown overnight in liquid medium were transferred to 2 mL centrifuge tubes and centrifuged at 12,000 rpm and 4 °C for one minute. The pellet was retained for subsequent DNA extraction, utilizing a genomic extraction kit(Invitrogen, Carlsbad, CA, USA) appropriate for both bacterial and fungal DNA, following the manufacturer’s guidelines. PCR amplification was then performed on the extracted DNA. Bacterial primers 27F (5′-AGAGTTTGATCCTGGCTCAG-3′) and 1492R (5′-GGTTACCTTGTTACGACTT-3′), and fungal primers ITS1 (5′-TCCGTAGGTGAACCTGCGG-3′) and ITS4 (5′-TCCTCCGCTTATTGATATGC-3′) were utilized. Post-amplification, a 1% agarose gel was prepared for electrophoresis, conducted at 110 V for 35 min in 1× TBE buffer, employing DS2000 as a molecular weight marker to ascertain bacterial target bands at approximately 1500 bp and fungal bands between 500 and 750 bp. Gel images were captured using a gel documentation system.

In the final stage, purification and sequencing of PCR products were performed. The obtained sequences were submitted to GenBank for BLAST3.2 analysis to identify species with high homology, completing the process of microbial identification and characterization in a detailed and scientifically rigorous manner.

### 2.8. Microbial Transformation of 4-VG Related Compounds

For the bacterial culture medium containing ferulic acid, a 100 mL solution of Luria–Bertani (LB) broth was prepared according to the manufacturer’s instructions. Ferulic acid (30 mg) was then dissolved in 1 mL of dimethyl sulfoxide (DMSO) and this solution was then transferred to the LB broth to create a final concentration of 0.3 g/L ferulic acid in the medium. The same procedure was followed to prepare a 0.4 g/L 4-VG bacterial culture medium. The bacterial strains were firstly inoculated into a 50 mL centrifuge tube containing 20 mL of the LB broth and incubated on a shaker (180 rpm, 37 °C, pH = 7, IKA, Staufen, Germany) for 18 h. 1.0 mL of the resulting medium was transferred to a 50 mL centrifuge tube containing 10 mL of the previously prepared culture medium with 0.3 g/L ferulic acid. The mixture was incubated on a shaker (180 rpm, 37 °C, pH = 7) for 72 h. The culture medium was then filtered out, and the transformed solution was subjected to three cycles of freeze-thawing followed by high-speed centrifugation (10,000 rpm, 10 min, Hamburg, Germany). The supernatant was collected and filtered through a 0.22 μm microporous membrane for further analysis.

For the preparation of the fungal culture medium with ferulic acid, a Yeast Peptone Dextrose (YPD) broth was prepared as directed by the manufacturer. 30.0 mg of ferulic acid was dissolved in 1 mL of DMSO, and this solution was added to 100 mL of YPD broth to achieve a final concentration of 0.3 g/L. Similarly, a 0.4 g/L 4-VG fungal culture medium was prepared using the same method. The fungal strain was firstly inoculated onto a YPD solid agar plate and incubated in a constant temperature incubator (30 °C, pH = 7, Thermo Fisher Scientific, Waltham, MA, USA) for 36 h. The ferulic acid fungal culture medium (10 mL) was placed into a 50 mL centrifuge tube, and a monoculture was scraped off from the solid agar plate and added into the tube. The culture was incubated on a shaker (180 rpm, 37 °C, pH = 7) for 4.5 days. The culture medium was filtered out, and the transformed solution was prepared following the method described above.

The content of ferulic acid, 4-vinylguaiacol, vanillin, and guaiacol were determined by the same HPLC-UV (detector: UVD) described in Section 2.5.

### 2.9. Statistical Analysis

All data was analyzed using GraphPad Prism 9.0 (GraphPad Software, San Diego, CA, USA). All experimental data was expressed as mean ± SDs of at least three biological replicates. The statistical significance of the differences between various treatments was measured by one-way ANOVA with Bonferroni post-test, considering *p* < 0.05 as a statistically significant difference.

## 3. Results

### 3.1. Analysis of CRCP Volatile Oil

**Figure 1:**
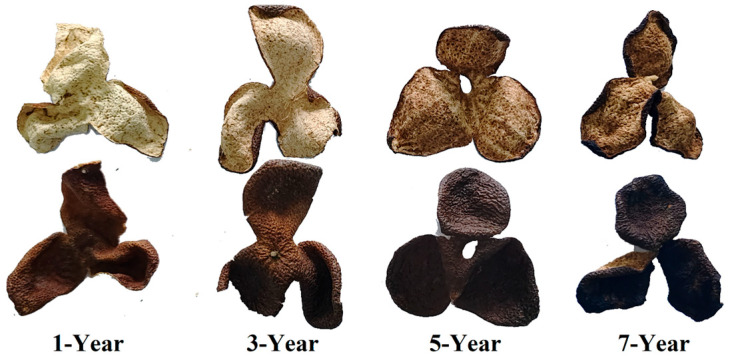
Morphological observation of CRCP in different aging years.

Volatile oil is one of the primary pharmacologically active components in CRCP [30] and plays a pivotal role in its aging process. In the current study, volatile oils from CRCP samples of different aging periods (1-Year, 3-Year, 5-Year, and 7-Year CRCP, as shown in Figure 1) were extracted through steam distillation and then analyzed for their composition and relative content using GC-MS. As listed in Table 1, a total of 29 components were identified, comprising 15 monoterpenes, 6 sesquiterpenes, 5 aromatics, 2 fatty aldehydes, and 1 alkaloid. Among these, the monoterpenes D-limonene and *γ*-terpinene were found to be the major components of CRCP volatile oil [7]. Additionally, the alkaloid methyl 2-(methylamino)benzoate, the monoterpenes β-thujene, α-pinene, β-pinene and β-myrcene, and one sesquiterpene α-sinensal showed higher contents compared to other minor components. Notably, the aromatic compound 4-VG demonstrated a trend of increasing relative content with the aging years.

### 3.2. Glucose Uptake Enhancing Activities of 19 Components in CRCP Volatile Oil

To assess the impact of CRCP volatile oil components on energy metabolism, which may potentially link to its therapeutic effects on gastrointestinal diseases and traditional massage treatments for abdominal pain, an insulin-stimulated glucose uptake model was used with C2C12 myotubes [24]. Skeletal muscle is critical for glucose metabolism and is a primary target of insulin [31]. Insulin resistance in skeletal muscle is a key factor in the impairment of insulin function, and reduced glucose uptake capacity in skeletal muscle significantly contributes to the development and progression of type 2 diabetes [32].

**Figure 2:**
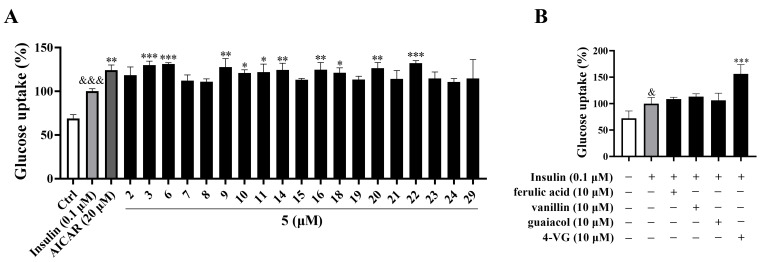
(**A**) Glucose consumption efficacy of 19 compounds from CRCP volatile oil. (**B**) 4-VG related metabolites B (& *p* < 0.05, &&& *p* < 0.001 vs. control group; * *p* < 0.05, ** *p* < 0.01, *** *p* < 0.001 vs. insulin group. Data are shown as mean ± S.D., *n* = 3).

In the glucose uptake assay, 19 components of CRCP volatile oil were tested, including α-pinene (**2**), β-pinene (**3**), β-myrcene (**6**), D-limonene (**7**), γ-terpinene (**8**), terpinolene (**9**), terpinen-4-ol (**10**), linalool (**11**), α-terpineol (**14**), decanal (**15**), carveol (**16**), perillaldehyde (**18**), thymol (**19**), p-thymol (**20**), carvacrol (**21**), 4-VG (**22**), methyl 2-(methylamino)benzoate (**23**), β-caryophyllene (**24**), and α-sinensal (**29**). Among these, β-pinene (**3**), β-myrcene (**6**), and 4-VG (**22**) significantly increased the insulin-stimulated uptake of glucose in C2C12 cells at a concentration of 5 μM (*p* < 0.001), showing greater potency than the positive control 5-aminoimidazole-4-carboxamide ribonucleotide (AICAR) at 20 μM (Figure 2A). Additionally, terpinolene (**9**), α-terpineol (**14**), carveol (**16**), and p-thymol (**20**) also demonstrated promising activities for glucose metabolism. These components may contribute to the metabolic-enhancing properties of CRCP volatile oils.

4-VG displayed the most remarkable efficacy in the glucose uptake assay, making it a prime candidate for further investigation. This compound, known for its unique flavor and potential bioactivity, has garnered significant interests in both food science and pharmacological research [33,34]. It is notably found in various beverages and foods, including beer, wine, and certain spices [35]. The biosynthesis of 4-VG occurs via the decarboxylation of ferulic acid by specific strains of yeast and bacteria during fermentation [35,36]. Furthermore, 4-VG can be further degraded by microbes to form vanillin and guaiacol [37]. Therefore, the glucose uptake enhancing capacities of four compounds involved in the microbial transformation pathway of 4-VG, including ferulic acid, 4-VG, vanillin, and guaiacol, were simultaneously assessed. Among them, 4-VG exhibited the most potent glucose uptake-enhancing activity (Figure 2B), providing practical evidence supporting the role of CRCP aging in enhancing biological activity.

### 3.3. 4-VG Enhanced Glucose Uptake in C2C12 Myotubes via Activating IRS-1/Akt/GSK-3β Signaling Pathway

**Figure 3:**
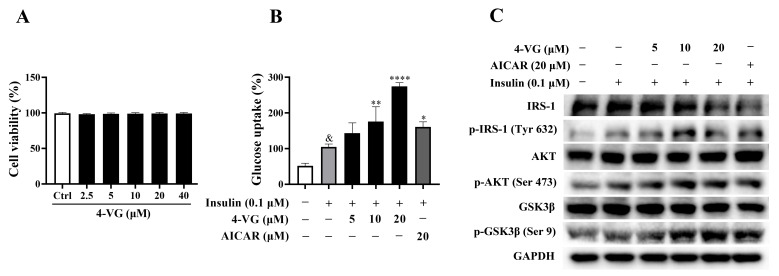
4-VG increased insulin-stimulated glucose uptake in C2C12 myotubes. (**A**) Cytotoxicity of 4-VG on C2C12 myotubes detected by MTT assay. (**B**) 4-VG enhanced insulin-stimulated glucose uptake in a dose-dependent manner in C2C12 myotubes. (**C**) 4-VG activated insulin signaling pathway in C2C12 myotubes. The expression of p-IRS-1, IRS-1, p-Akt, Akt, p-GSK3β, and GSK3β was analyzed by Western blotting. GAPDH was used as house-keeping protein. Data are presented as the mean ± SD; *n* = 3. & *p* < 0.05 vs. control group. * *p* < 0.05, ** *p* < 0.01 **** *p* < 0.0001 vs. insulin group.

First, cytotoxicity of 4-VG was assessed using the MTT assay, which showed no significant cytotoxic effects on C2C12 myotubes at concentrations up to 40 μM (Figure 3A). Furthermore, 4-VG increased glucose uptake in C2C12 myotubes in a dose-dependent manner. At a concentration of 20 μM, 4-VG significantly enhanced insulin-mediated glucose uptake levels, achieving approximately 275% of the baseline level, compared to 160% with 20 μM AICAR (Figure 3B).

To further explore the underlying mechanisms, Western blot techniques were employed to detect representative proteins in the insulin signaling pathway. As depicted in Figure 3C, insulin stimulation led to the phosphorylation of insulin receptor substrate 1 (IRS-1), protein kinase B (Akt), and glycogen synthase kinase 3β (GSK-3β). Notably, treatment with 4-VG further increased the phosphorylation levels of these proteins. These results collectively suggest that 4-VG enhances insulin-stimulated glucose uptake in C2C12 myotubes by activating the IRS-1/Akt/GSK-3β signaling pathway.

### 3.4. 4-VG Exhibited Anti-Diabetic Effects on an HFD/STZ-Induced Diabetic Mouse Model

**Figure 4:**
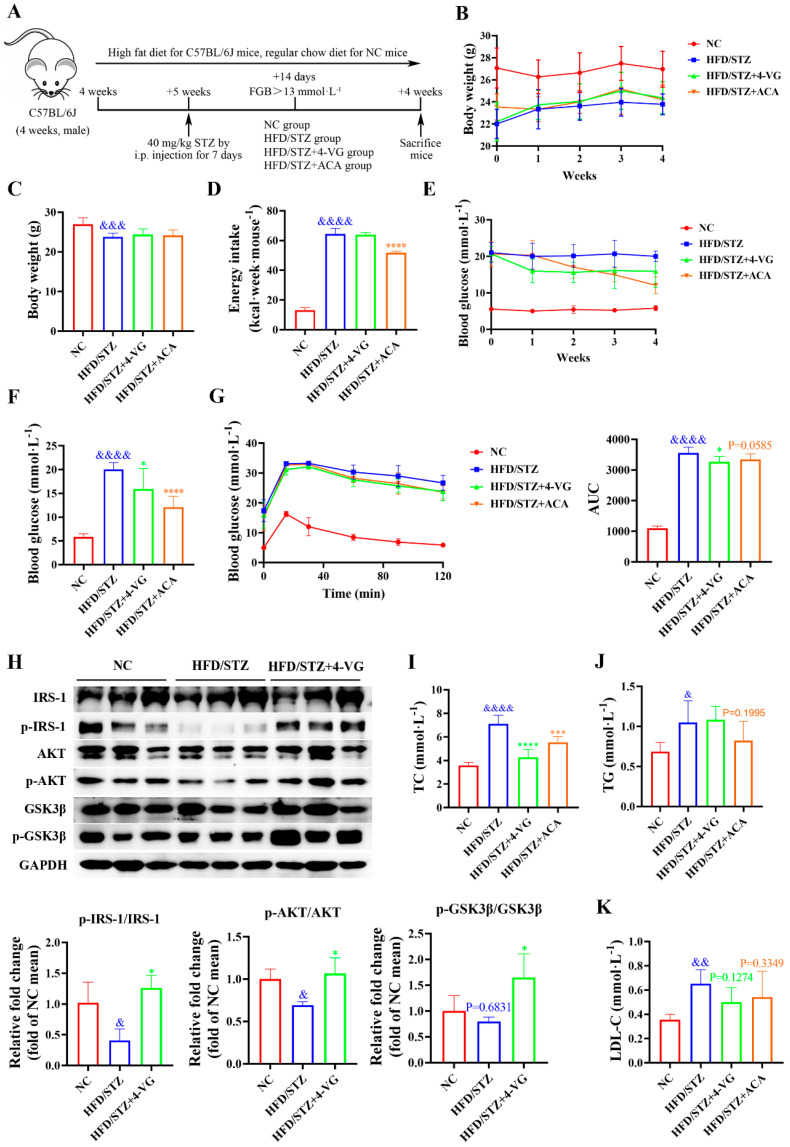
4-VG ameliorated insulin resistance in HFD/STZ-induced diabetic mice. (**A**) The experimental procedure of HFD/STZ-induced diabetic mice; (**B**) Body weight of mice under RD or HFD feeding; (**C**) Body weight of mice after four-week intervention of 4VG; (**D**) Weekly energy intake per mouse; (**E**) Fasting blood glucose of mice; (**F**) Fasting blood glucose of mice after four-week intervention with 4-VG; (**G**) Glucose tolerance test was performed after four-week 4-VG treatment. AUC of each group was calculated; (**H**) The expression of key proteins in insulin signaling pathway in gastrocnemius muscle was determined by Western blot; (**I**) Total cholesterol (TC) in serum; (**J**) Total glycerides (TG) in serum; (**K**) LDL cholesterol (LDL-C) in serum. Data are shown as mean ± SD. (*n* = 6~7). &/* *p* < 0.05, && *p* < 0.01, &&&/*** *p* < 0.001, &&&&/**** *p* < 0.0001 vs. normal control and model group, respectively.

To further validate the anti-diabetic efficacy of 4-VG, an HFD/STZ-induced diabetic mice model was utilized. The experimental procedure was outlined in Figure 4A, with acarbose (ACA) at a dose of 50 mg/kg used as a positive control. The results showed that mice in the HFD/STZ group experienced significant weight loss. After four weeks of intervention with 4-VG and ACA, there was a slight increase in body weight compared to the untreated HFD/STZ mice (Figure 4B,C). Energy intake was significantly elevated in the HFD/STZ group, and ACA treatment notably reduced this trend, while 4-VG intervention resulted in only a slight decrease (Figure 4D).

**Figure 5:**
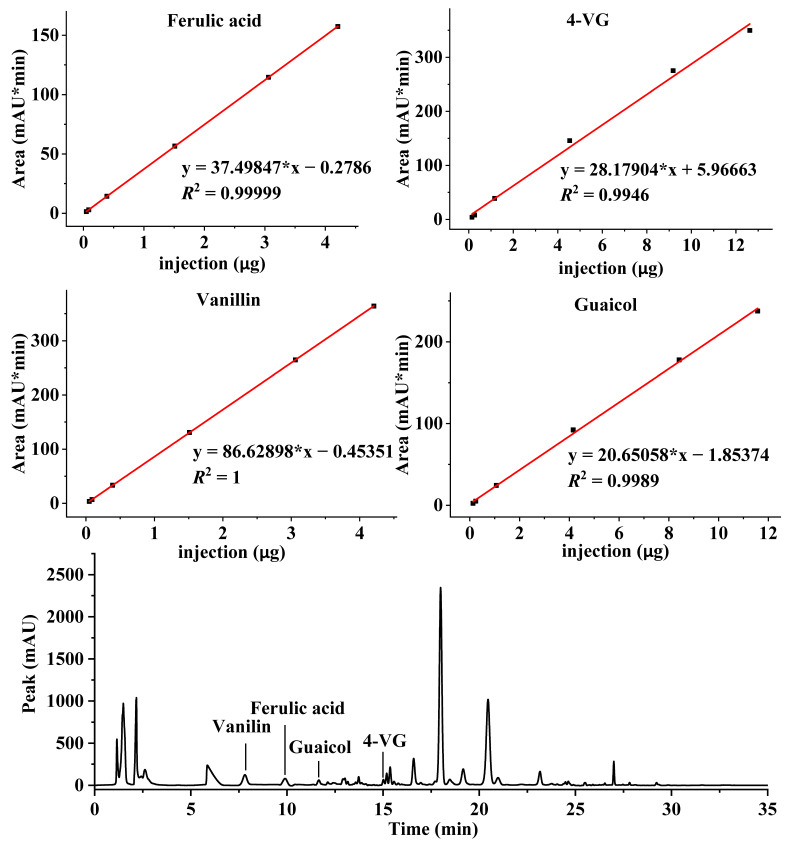
HPLC-UV determination of the contents of ferulic acid, 4-vinylguaiacol, vanillin, and guaiacol in 5-Year CRCP. The standard curves of four compounds and HPLC chromatogram of CRCP extract.

Fasting blood glucose levels were monitored weekly throughout the intervention. As shown in Figure 5, the fasting blood glucose of diabetic mice significantly decreased after just one-week of 4-VG treatment, while a reduction was observed only after two weeks of ACA treatment. After four weeks, 4-VG reduced fasting blood glucose by approximately 21% (Figure 4F). As the intervention continued, the hypoglycemic effect of 4-VG stabilized, suggesting 4-VG may help prevent the risk of hypoglycemia after intervention, while ACA further decreased fasting blood glucose. During the glucose tolerance test (GTT), the glucose disposal rate was markedly impaired in the HFD/STZ group, but 4-VG treatment significantly improved glucose clearance, akin to ACA. Notably, 4-VG demonstrated a stronger hypoglycemic response to oral glucose administration than ACA (Figure 4G). Additionally, Western blot analysis of insulin pathway proteins in skeletal muscle showed that 4-VG treatment reversed the suppression of IRS-1, AKT, and GSK-3β phosphorylation induced by HFD/STZ (Figure 4H).

The serum lipid profile was evaluated. Total cholesterol (TC) levels were significantly elevated in the HFD/STZ mice, but 4-VG treatment effectively reduced TC levels, showing a stronger effect than ACA (Figure 4I). However, 4-VG did not significantly affect serum triglycerides (TG) levels (Figure 4J), aligning with existing data on CRP extracts [38]. Additionally, 4-VG partially restored low-density lipoprotein cholesterol (LDL-C) levels (Figure 4K).

In conclusion, 4-VG was identified as an effective anti-diabetic agent by increasing insulin-stimulated glucose uptake through IRS-1/AKT/GSK-3β signaling pathway in C2C12 myotubes. In the HFD/STZ-induced diabetic mice model, 4-VG effectively alleviated the symptoms of hyperglycemia and improved lipid metabolism via activating the insulin signaling pathway. These findings provided a scientific basis for the potential development 4-VG and CRCP as functional foods or pharmaceutical products.

### 3.5. HPLC-UV, Metabolomics and Microbiome Analysis of 4-VG Related Compounds in CRCP

The quality and efficacy of CRCP are known to improve with aging, likely due to changes in compound composition and the microbial community [39]. The previous bioactivity study highlighted the potential role of 4-VG as a crucial active component in CRCP volatile oil, suggesting its involvement as a key intermediate in CRCP aging. To further investigate this, the contents of four metabolic-related compounds, including ferulic acid, 4-VG, vanillin, and guaiacol, were simultaneously determined by HPLC-UV (Figure 5). The results showed that the content of 4-VG exhibited an obviously increasing trend in 1, 3, 5, 7-year CRCP. In contrast, the content of ferulic acid gradually decreased after aging. In addition, vanillin and guaiacol in CRCP also increased with the extension of aging time (Table 2). These findings, along with literature reports [35,36,37], suggest a microbial transformation process, indicating that microorganisms may play a significant role in promoting the aging of CRCP.

**Figure 6:**
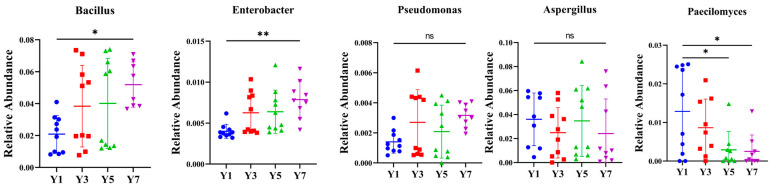
The abundance of related bacteria and fungus in genus level in CRCP of different years (*n* = 10). * *p* < 0.05 and ** *p* < 0.01 vs. Y1 group.

**Figure 7:**
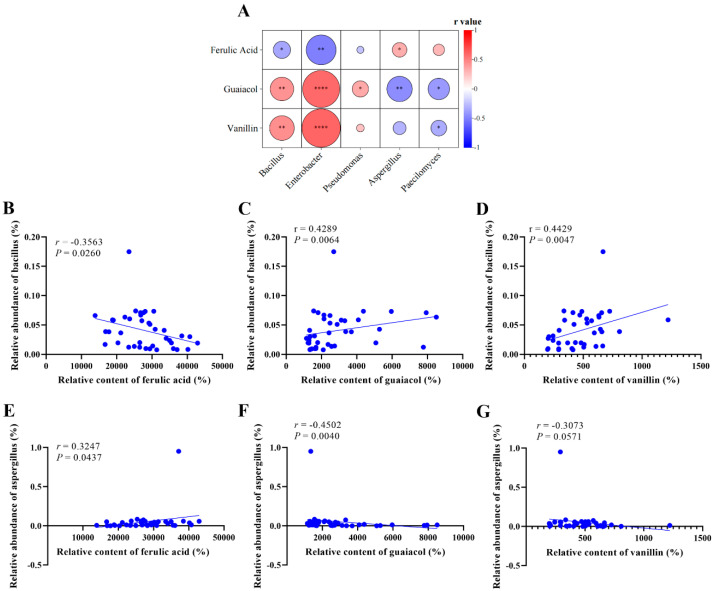
The correlations between the storage time dependently altered metabolites and bacteria and fungus in CRCP of different years were analyzed by using nonparametric Spearman correlation (*n* = 10). (**A**) The correlations between metabolites and bacteria and fungus. The correlation between *Bacillus* and ferulic acid (**B**), guaiacol (**C**), vanillin (**D**). The correlation between *Aspergillus* and ferulic acid (**E**), guaiacol (**F**), vanillin (**G**). * *p* < 0.05, ** *p* < 0.01 and **** *p* < 0.0001.

To gain further insights, a microbiome analysis of CRCP was accomplished using Illumina Miseq sequencing. The results demonstrated an increasing trend in the abundance of the *Bacillus* and *Enterobacter* genera over time, while the abundance of the *Paecilomyces* genus decreased with aging (Figure 6). The correlations between age-related changes in metabolites and microbial communities were analyzed using nonparametric Spearman correlation. The relative abundances of *Bacillus* and *Enterobacter* were negatively correlated with the relative content of ferulic acid, andpositively correlated with the relative contents of guaiacol and vanillin, which are microbial transformation products of ferulic acid (Figure 7A–D). Although the relative abundance of *Aspergillus fumigatus* did not significantly change with the increased storage time of CRCP, it was positively correlated with the relative content of ferulic acid and negatively correlated with the relative content of guaiacol (Figure 7A,E–G). These findings suggested that the decrease in ferulic acid and the increase in guaiacol and vanillin with aging may be attributed to microbial transformation.

### 3.6. Isolation and Identification of Microorganisms from CRCP, and In Vitro Microbial Transformation Experiments

Three abundant microbial strains were isolated from CRCP by streak plate method, including two bacterial strains and one fungal strain. These microorganisms were identified tthrough16S rRNA and ITS rRNA sequencing as *Priestia aryabhattai*, *Bacillus velezensis*, and *Aspergillus fumigatus*.

**Scheme 1:**
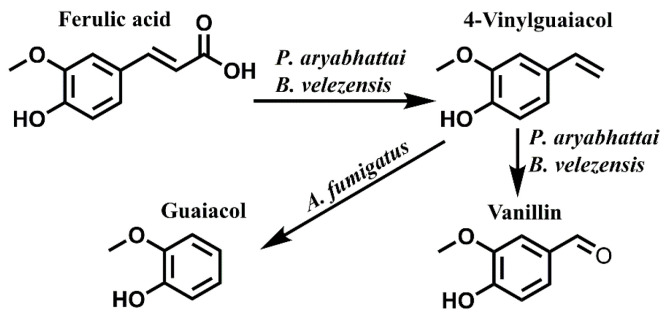
The biotransformation pathway from ferulic acid to guaiacol under the influence of CRCP microorganisms.

To investigate the microbial transformation capabilities of these strains, in vitro microbial transformation experiments wer accomplished using ferulic acid and 4-VG as substrates (Table 3, Scheme 1).

The results showed that ferulic acid could be transformed into 4-VG by *P. aryabhattai* and *B. velezensis* with molar yields of 69.58% and 31.65%, respectively. Moreover, the two bacterial strains were capable of converting 4-VG into vanillin with a molar yield about 2.315%. The fungal strain, *A. fumigatus*, exhibited the ability to directly convert 4-VG into guaiacol with a molar yield of 6.665%. These transformation experiments, conducted with CRCP endophytes, confirmed the involvement of these key microorganisms in transforming active ingredients during CRCP’s aging process. The findings not only validated the transformation pathway from ferulic acid to 4-VG and subsequently to vanillin and guaiacol but also provided clear evidence of the chemical changes occurring during the aging process of CRCP.

## 4. Discussion

Although CRP extracts have been reported for their therapeutic effects on endothelial dysfunction and vascular inflammation in diabetic rats [40], as well as treatment of type 2 diabetic osteoporosis [41], the specific anti-diabetic component of CRP have not been thoroughly identified. The current study elucidates the complex aging process of CRCP through anti-diabetic activity-driven assays and detailed research on a specific crucial compound, 4-VG, highlighting its increasing role as a metabolite in CRCP and its potential as a therapeutic agent for diabetes.

Metabolomics and microbiome analyses offer new insights into the symbiotic relationship between microbial activity and the chemical evolution of CRCP. The microorganisms identified—*P. aryabhattai*, *B. velezensis*, and *A. fumigatus*—play a crucial role in converting ferulic acid into the more active compound 4-VG. This transformation of the non-volatile compound ferulic acid into the volatile component 4-VG by specific microorganisms enriches the diversity of the volatile oil profile in CRCP and is likely contributes to the gradual mellowing of its properties during aging. Therefore, the content of 4-VG could serve as an index for assessing CRCP aging. This underscores the importance of microbial biotransformation in enhancing the quality of herbal medicines, a concept that could be applied within the CRCP industry and extended to other medicinal plants. Understanding these complex microbial interactions could lead to innovative strategies for enhancing the efficacy of herbal medicines and accelerating the aging process, potentially through targeted modulation of the microbiome.

4-VG is a phenolic compound widely recognized as a key flavor and aroma molecule in various foods and beverages [42]. Beyond its sensory properties, emerging research has gradually revealed significant therapeutic and biological activities. The phenolic nature of 4-VG conferred its potent antioxidant properties [43], indicating it could be used to prevent aging, metabolic disorder, cardiovascular disease, etc. In addition, 4-VG was demonstrated to possess obvious anti-inflammatory potential. Asami revealed 4-VG suppressed the expression of NO and iNOS, induced Keap1 degradation and Nrf2 translocation [44]. This suggested promising application of 4-VG in managing inflammatory disease like rheumatoid arthritis. Moreover, preliminary research indicated that 4-VG may possess chemopreventive and anticancer properties. Luo reported 4-VG has been shown to chemotherapy enhancement, apoptosis induction and cell cycle arrest in HCT-116 and HT-29 cells [45]. Lee confirmed 4-VG mitigated cholangiocarcinoma cell malignancy [46]. Furthermore, 4-VG exhibited good antimicrobial activity. Yang demonstrated 4-VG showed antifungal activity against pathogen *Fusarium oxysporum* [47] and antibacterial activity against pathogen *Vibrio cholerae* [48]. These findings also provided strong support for CRCP as a functional food.

4-VG is a common compound in our daily diet, widely present in the food and spice industries [35]. Besides its potential therapeutic effects on disease, such as anti-tumor metastasis [34], the impact of 4-VG on human metabolism may be a critical area of interest. Our research has identified 4-VG as a potent enhancer of glucose metabolism, highlighting its therapeutic potential and providing insight into the mechanisms underlying the health benefits of commonly consumed dietary components.

## 5. Conclusions

This study utilized chemical analysis, molecular biology, and microbiology techniques to deconstruct the complex aging process of CRCP through the examination of a representative 4-VG metabolic pathway. Drawing inspiration from the traditional Chinese medicine concept of “reducing the pungent and hot odor nature of CRP” during aging, the components of the volatile oil of CRCP and their glucose metabolism-enhancing activities were studied. 4-VG was identified as a crucial active component in the volatile oil, closely linked to the aging process of CRCP. It not only showed an increasing trend in relative percentage within the volatile oil as aging progressed, but also significantly enhanced glucose metabolism in both C2C12 myotubes and an HFD/STZ-induced diabetic mice model by elevating the phosphorylation levels of insulin pathway-related proteins. Furthermore, the content of four compounds related to 4-VG metabolism in CRCP, namely ferulic acid, 4-VG, vanillin, and guaiacol, were quantitatively determined in 5-Year CRCP. Metabolomics analysis confirmed changes in the relative contents of ferulic acid, vanillin, and guaiacol throughout the aging process. The potential related microbial genus was analyzed simultaneously by metabolomics study. Additionally, three microorganisms were isolated and identified from CRCP, and their metabolic transformations of the four components were investigated. These findings provide evidence that microorganisms play a role in promoting the metabolic pathways from ferulic acid to 4-VG, and from 4-VG to vanillin and guaiacol, offering valuable insights into the aging process of CRCP.

Overall, this study not only identified the key active component and its mechanism of action on glucose metabolism in CRCP’s volatile oil but also provided novel experimental evidence for understanding the aging process of CRCP. There are still some limitations in the current research. The biological targets and binding sites of 4-VG have not been fully elucidated. Due to the limited number of microorganisms isolated and studied, the complex dynamics of 4-VG transformation during the aging of CRCP may not be fully represented.

## Figures and Tables

**Table 1:** Chemical compositions of the volatile oils of CRCP with different aging years.

No.	Component	Retention Time (min)	Structure Type	Relative Content (%)			
				1-Year	3-Year	5-Year	7-Year
**1**	*β*-Thujene	3.986	monoterpene	1.38	0.75	1.38	1.34
**2**	*α*-Pinene	4.001	monoterpene	3.44	2.18	3.65	3.38
**3**	*β*-Pinene	4.145	monoterpene	3.23	2.26	3.13	3.04
**4**	*α*-Phellandrene	4.269	monoterpene	0.06	0.03	0.11	0.06
**5**	*α*-Terpinene	4.689	monoterpene	0.45	0.42	0.46	0.4
**6**	*β*-Myrcene	4.822	monoterpene	1.26	0.65	0.72	1.51
**7**	D-Limonene	5.583	monoterpene	59.09	58.51	59.88	59.74
**8**	*γ*-Terpinene	6.179	monoterpene	17.27	18.20	17.85	17.47
**9**	Terpinolene	6.816	monoterpene	1.06	0.85	0.91	0.75
**10**	Terpinen-4-ol	8.893	monoterpene	0.64	0.67	0.56	0.54
**11**	Linalool	7.003	monoterpene	0.43	0.3	0.11	0.21
**12**	Nonanal	7.136	fatty aldehyde	0.07	0.11	0.05	0.06
**13**	*p*-Cymen-8-ol	9.050	aromatic compound	0.03	0.03	0.03	0.03
**14**	*α*-Terpineol	9.200	monoterpene	0.87	0.76	0.47	0.59
**15**	Decanal	9.500	fatty aldehyde	0.28	0.38	0.22	0.24
**16**	Carveol	9.875	monoterpene	0.03	0.04	-	0.01
**17**	Citronellol	10.036	monoterpene	0.12	0.05	0.04	0.08
**18**	Perillaldehyde	11.292	monoterpene	0.24	0.16	0.08	0.12
**19**	Thymol	11.476	aromatic compound	0.96	2.25	0.92	0.57
**20**	*p*-Thymol	11.633	aromatic compound	0.63	0.54	0.26	0.47
**21**	Carvacrol	11.884	aromatic compound	0.08	0.17	0.06	0.04
**22**	4-Vinylguaiacol	12.240	aromatic compound	0.11	0.26	0.41	0.34
**23**	Methyl 2-(methylamino)benzoate	14.613	alkaloid	1.51	1.01	1.3	0.78
**24**	*β*-Caryophyllene	14.961	sesquiterpene	0.19	0.26	0.18	0.15
**25**	*α*-Farnesene	16.987	sesquiterpene	0.63	0.58	0.75	0.69
**26**	*δ*-Cadinene	17.398	sesquiterpene	0.09	-	0.08	0.09
**27**	*α*-Selinene	16.776	sesquiterpene	0.04	0.07	-	0.04
**28**	*γ*-Selinene	19.756	sesquiterpene	-	0.05	0.05	0.06
**29**	*α*-Sinensal	20.769	sesquiterpene	1.01	2.98	1.87	1.09

**Table 2:** The content of ferulic acid, 4-VG, vanillin and guaiacol in CRCP with different aging years.

Component	Content (mg/g) ^a^			
	1-Year	3-Year	5-Year	7-Year
Ferulic acid	0.3998 ± 0.0311	0.2994 ± 0.0579	0.2863 ± 0.0527	0.2201 ± 0.0088
4-VG	0.0154 ± 0.0037	0.0375 ± 0.0025	0.0369 ± 0.0023	0.0446 ± 0.0020
Vanillin	0.0177 ± 0.0009	0.0432 ± 0.0136	0.0466 ± 0.0063	0.0617 ± 0.0088
Guaiacol	0.8952 ± 0.0636	0.9430 ± 0.0548	1.0060 ± 0.0604	1.0277 ± 0.0747

^a^ Content values were expressed as mean ± SD (*n* = 3).

**Table 3:** Microbial transformations of ferulic acid and 4-VG.

Substrate (C, mg/10 mL)	Metabolite (C, mg/10 mL)	Strain	Time (h)	Mol. Yield (%)
Ferulic acid (0.3)	4-VG (1.614)	*P. aryabhattai*	72	69.58
	4-VG (0.7343)	*B. velezensis*	72	31.65
4-VG (0.4)	Vanillin (0.09384)	*P. aryabhattai*	120	2.315
	Vanillin (0.1010)	*B. velezensis*	120	2.491
	Guaiacol (0.2204)	*A. fumigatus*	120	6.665

## Data Availability

The original contributions presented in this study are included in the article/Supplementary Materials. Further inquiries can be directed to the corresponding author.

## Supplementary Materials

The following supporting information can be downloaded at: https://www.mdpi.com/article/10.3390/foods14203489/s1, S1: Methods for HPLC-UV analysis of the contents of ferulic acid, 4-vinylguaiacol, vanillin, and guaiacol in CRCP; Figure S1: HPLC chromatogram of mixed standards of vanillin, ferulic acid, guaiacol, and 4-VG; S3: Bacterial and fungal transformation experiments; S4: Identification of three microbials isolated from CRCP.

## Abbreviations

The following abbreviations are used in this manuscript:

CRCP	Citri Reticulatae ‘Chachiensis’ Pericarpium
4-VG	4-Vinylguaiacol
HFD	High fat diet
ACA	Acarbose
