# Supplementary material for: 4-Vinylguaiacol in Citri Reticulatae ‘Chachiensis’ Pericarpium Volatile Oil: A Microbial-Mediated Aging Marker Enhances Glucose Metabolism
Source: Foods. 2025 Oct 14;14(20):3489. doi: 10.3390/foods14203489 (PMC12563654; doi:10.3390/foods14203489)
Supplement: Supplementary file 1 [file foods-14-03489-s001.zip › foods-3798682-supplementary.pdf]

Supplementary Materials

for

4-Vinylguaiacol in Citri Reticulatae 'Chachiensis' Pericarpium Volatile Oil: A Microbial-Mediated Aging Marker Enhances Glucose Metabolism

*Corresponding authors.

School of Pharmaceutical Sciences, Zhejiang Chinese Medical University, Hangzhou 311402, China. E-mail: lsgan@zcmu.edu.cn (Prof. Li-She Gan), ligenl@um.edu.mo (Prof. Li-Gen Lin), wyuchemwrh@126.com (Prof. Ri-Hui Wu)

S1. Methods for HPLC-UV analysis of the contents of ferulic acid, 4-vinylguaiacol, vanillin, and guaiacol in CRCP.

2.5 g of 5-Year CRCP samples were accurately weighed and transferred into a 25 mL volumetric flask. A methanol-ethyl acetate (5:1) mixed solution was added to the mark, followed by sonication for 20 minutes. After cooling, the volume was made up to the mark with the methanol-ethyl acetate (5:1) mixed solution and mixed well. Prior to analysis, the solution was filtered through a 0.22 μm PTFE membrane into a 2 mL sample vial for analysis.

Standard Curve Preparation

Vanillin (0.1 g), ferulic acid (0.1 g), guaiacol (0.5 g), and 4-VG (0.3 g) were accurately weighed separately and dissolved in methanol in a 100 mL volumetric flask to obtain standard stock solutions. Then, 5 mL of the vanillin stock solution, 5 mL of the ferulic acid stock solution, 1 mL of the guaiacol stock solution, and 5 mL of the 4-VG stock solution were pipetted into a 25 mL volumetric flask and diluted to volume with methanol. A series of standard solutions for the corresponding compounds were prepared by stepwise dilution to establish the standard curves. The concentration ranges for the standard curves were as follows: vanillin: 0.004, 0.008, 0.016, 0.032, 0.08, 0.2 mg/mL; ferulic acid: 0.004, 0.008, 0.016, 0.032, 0.08, 0.2 mg/mL; guaiacol: 0.2, 0.4, 0.8, 1.6, 4, 10 mg/mL; 4-VG: 0.012, 0.024, 0.048, 0.096, 0.24, 0.6 mg/mL. Prior to analysis, the solutions were filtered through a 0.22 μm PTFE membrane into 2 mL sample vials for analysis.

Chromatographic Conditions

Chromatographic system: Waters Acquity Arc High-Performance Liquid Chromatography (HPLC); column: Cyncronis C18 (150 mm × 4.6 mm, 5 μm);

Column temperature: 35°C; Mobile phase: 0.1% phosphoric acid in water (A) - acetonitrile (B); Gradient elution: 0–6 min, 15% B; 6–10 min, 15–35% B; 10–12 min, 35–50% B; 12–20 min, 50% B; 20–25 min, 50–100% B; 25–30 min, 100% B; Flow

rate: 1.0 mL/min; Detection wavelength: 230 nm; Injection volume: 10 μL for the test solution, and 0.12, 0.22, 0.97, 3.78, 7.65, 10.52 μL for the reference standard solutions, respectively.

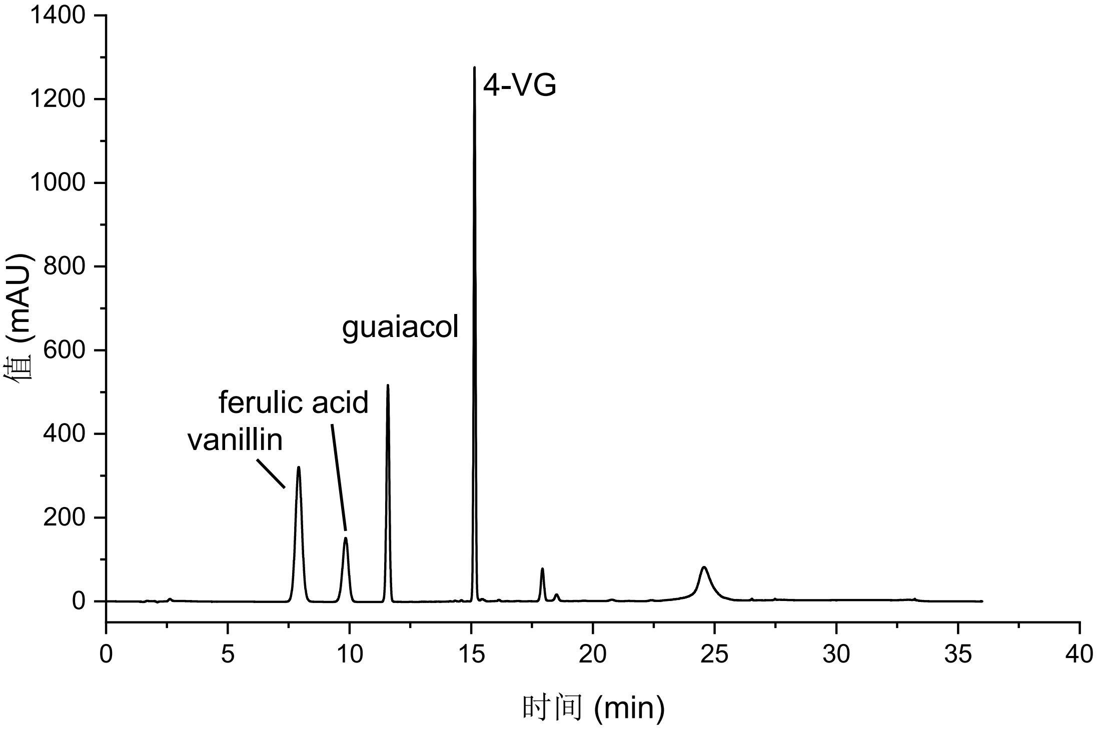

Figure S1 HPLC chromatogram of mixed standards of vanillin, ferulic acid, guaiacol, and 4-VG.

Calibration curves data:

ferulic acid					
injection	peak area	peak area	peak area	Mean	SD
(μ)g	mAU*min	mAU*min	mAU*min	mAU*min	mAU*min
0.048	1.44956	1.4588	1.3888	1.43239	0.03803
0.088	2.88402	2.98915	2.91656	2.92991	0.05382
0.388	14.33905	14.16151	14.40798	14.30284	0.12716
1.512	56.55425	56.56919	56.65288	56.59211	0.05316
3.060	114.5322	114.6568	114.6228	114.6039	0.06441
4.208	157.453	157.1967	157.4093	157.353	0.13713

vanillin					
injection	peak area	peak area	peak area	Mean	SD
(μ)g	mAU*min	mAU*min	mAU*min	mAU*min	mAU*min
0.048	3.54427	3.56936	3.43309	3.51557	0.07253
0.088	6.92976	7.13524	6.96497	7.00999	0.10989
0.388	33.42846	32.96985	33.55375	33.31735	0.3074
1.512	130.7257	130.79968	130.97167	130.83235	0.1262
3.060	264.46783	264.80777	264.67577	264.65046	0.17138
4.208	363.95099	363.71943	364.17734	363.94925	0.22896

4-VG					
injection	peak area	peak area	peak area	Mean	SD
(μ)g	mAU*min	mAU*min	mAU*min	mAU*min	mAU*min
0.144	4.30939	4.24625	3.98795	4.18119	0.17031
0.264	8.20421	8.44304	8.24136	8.2962	0.12851
1.164	39.0212	38.46076	39.14474	38.87556	0.36451
4.536	145.78363	145.82883	146.0202	145.87755	0.12558
9.180	275.01793	276.04707	275.15464	275.40655	0.55891
12.624	348.99047	349.61396	350.48354	349.69599	0.74991

guaiacol					
injection	peak area	peak area	peak area	Mean	SD
(μ)g	mAU*min	mAU*min	mAU*min	mAU*min	mAU*min
0.132	2.53498	2.54971	2.43674	2.50714	0.06142
0.242	5.00697	5.1421	5.02197	5.05701	0.07407
1.067	24.34269	24.00465	24.42976	24.25903	0.22456
4.158	92.08023	92.13463	92.30354	92.1728	0.11645
8.415	177.81696	177.9618	177.86934	177.8827	0.07334
11.572	237.77745	237.36104	237.69011	237.60953	0.21959

S3. Bacterial and fungal transformation experiments

3.1. Materials and Instruments

Materials: Xinhui Dried Tangerine Peel, Vanillin, Ferulic Acid, Guaiacol, 4-VG (4-Vinylguaiacol), Phosphoric Acid, Acetonitrile (HPLC grade),

Ethyl Acetate (Analytical grade), Methanol (Analytical grade), LB Medium, YPD Medium, Microporous Filter Membrane (Organic, 0.88μm, 0.45μm, 0.22μm), Syringe Filter (PTFE, 0.22μm).

3.2. Methods

3.2.1 Bacterial Transformation and Sample Preparation

A ferulic acid bacterial culture medium was prepared: LB medium solution was prepared according to the instructions. Precisely 30 mg of ferulic acid was weighed, dissolved in 1 mL of DMSO, and the solution was transferred to 100 mL of LB medium solution to prepare a 0.3 g/L ferulic acid bacterial culture medium. Using the same method, a 0.4 g/L 4-VG bacterial culture medium was also prepared.

20 mL of LB medium solution was added to a 50 mL centrifuge tube. Bacteria strain KH-2018-45(2) was inoculated into the centrifuge tube and cultured in a shaker (180 r/min, 37°C, pH=7) for 18 h.

10 mL of the ferulic acid bacterial culture medium was added to a 50 mL centrifuge tube. 1 mL of the cultured bacterial solution was added to the centrifuge tube and cultured in a shaker (180 r/min, 37°C, pH=7) for 72 h to obtain the transformation broth.

The medium was filtered out. After three freeze-thaw cycles and high-speed centrifugation (10,000 r/min, 10 min), the supernatant was passed through a 0.22 μm microporous filter membrane and then ready for analysis.

Using the same method described above, other bacteria were transformed to prepare ferulic acid bacterial transformation samples, as well as 4-VG bacterial transformation samples.

3.2.2 Fungal Transformation and Sample Preparation

A ferulic acid fungal culture medium was prepared: YPD medium solution was prepared according to the instructions. Precisely 30 mg of ferulic acid was weighed, dissolved in 1 mL of DMSO, and the solution was transferred to 100 mL of YPD medium solution to prepare a 0.3 g/L ferulic acid fungal culture medium. Using the same method, a 0.4 g/L 4-VG fungal culture medium was also prepared.

The fungal strain was inoculated onto a plate containing solid YPD medium and cultured in a constant temperature chamber (30°C, pH=7) for 36 h.

10 mL of the ferulic acid fungal culture medium was added to a 50 mL centrifuge tube. A single colony from the seed medium was selected, part of the mycelium was scraped and added to the centrifuge tube, and cultured in a shaker (180 r/min, 30°C, pH=7) for 108 h to obtain the transformation broth.

The medium was filtered out. Referring to the method in 2.1 for preparation, the ferulic acid fungal transformation sample for HF-2018-22, and the 4-VG fungal transformation samples for HF-2018-22 and KH-2020-36(2) were prepared.

3.2.3 Test Solution Preparation

Precisely 1.000 g of the powdered Dried Tangerine Peel sample was weighed and placed into a 10 mL volumetric flask. The flask was filled to the mark with a methanol-ethyl acetate (5:1) mixed solution, sonicated for 20 min, allowed to cool, then the methanol-ethyl acetate (5:1) mixed solution was added again to bring the volume back to the mark. The mixture was shaken well and passed through a 0.22 μm organic filter membrane, ready for analysis.

Reference Standard Solution Preparation: Precisely 0.004 g of vanillin, 0.004 g of ferulic acid, 0.011 g of guaiacol, and 0.012 g of 4-VG were weighed and placed into separate 10 mL volumetric flasks. Methanol was added to volume, and the solutions were passed through a microporous filter membrane, ready for analysis.

3.2.4 Liquid Chromatography Detection Method

Chromatographic column: Cyncronis C18 (150mm × 4.6mm, 5 μm); Column temperature: 35°C; Mobile phase: 0.1% phosphoric acid in water (A) - acetonitrile (B); Gradient elution: 0-6 min, 15% B; 6-10 min, 15-35% B; 10-12 min, 35-50% B; 12-20 min, 50% B; 20-25 min, 50-100% B; 25-30 min, 100% B; Flow rate: 1.0 mL/min; Detection wavelength: 230 nm; Injection volume: 10 μL for the test solution, and 0.12, 0.22, 0.97, 3.78, 7.65, 10.52 μL for the reference standard solutions, respectively.

S4. Identification of three microbials isolated from CRCP

(1) Priestia aryabhattai

16S rRNA sequencing:

GCGAACTGATTAGAAGCTTGCTTCTATGACGTTAGCGGCGGACGGGTGAGT
AACACGTGGGCAACCTGCCTGTAAGACTGGGATAACTTCGGGAAACCGAA
GCTAATACCGGATAGGATCTTCTCCTTCATGGGAGATGATTGAAAGATGGTT
TCGGCTATCACTTACAGATGGGCCCGCGGTGCATTAGCTAGTTGGTGAGGT
AACGGCTCACCAAGGCAACGATGCATAGCCGACCTGAGAGGGTGATCGGC
CACACTGGGACTGAGACACGGCCCAGACTCCTACGGGAGGCAGCAGTAGG
GAATCTTCCGCAATGGACGAAAGTCTGACGGAGCAACGCCGCGTGAGTGA
TGAAGGCTTTCGGGTCGTAAAACTCTGTTGTTAGGGAAGAACAAGTACGA
GAGTAACTGCTCGTACCTTGACGGTACCTAACCAGAAAGCCACGGCTAACT
ACGTGCCAGCAGCCGCGGTAATACGTAGGTGGCAAGCGTTATCCGGAATTA
TTGGGCGTAAAGCGCGCGCAGGCGGTTTCTTAAGTCTGATGTGAAAGCCC
ACGGCTCAACCGTGGAGGGTCATTGGAAACTGGGGAACTTGAGTGCAGAA
GAGAAAAGCGGAATTCCACGTGTAGCGGTGAAATGCGTAGAGATGTGGAG
GAACACCAGTGGCGAAGGCGGCTTTTTGGTCTGTAACTGACGCTGAGGCG
CGAAAGCGTGGGGAGCAAACAGGATTAGATACCCTGGTAGTCCACGCCGT
AAACGATGAGTGCTAAGTGTTAGAGGGTTTCCGCCCTTTAGTGCTGCAGCT
AACGCATTAAGCACTCCGCCTGGGGAGTACGGTCGCAAGACTGAAACTCA
AAGGAATTGACGGGGGCCCGCACAAGCGGTGGAGCATGTGGTTTAATTCG
AAGCAACGCGAAGAACCTTACCAGGTCTTGACATCCTCTGACAACTCTAG
AGATAGAGCGTTCCCCTTCGGGGGACAGAGTGACAGGTGGTGCATGGTTG
TCGTCAGCTCGTGTCGTGAGATGTTGGGTTAAGTCCCGCAACGAGCGCAAC
CCTTGATCTTAGTTGCCAGCATTTAGTTGGGCACTCTAAGGTGACTGCCGGT
GACAAACCGGAGGAAGGTGGGGATGACGTCAAATCATCATGCCCCTTATGA
CCTGGGCTACACACGTGCTACAATGGATGGTACAAAGGGCTGCAAGACCG
CGAGGTCAAGCCAATCCCATAAAACCATTCTCAGTTCGGATTGTAGGCTGC
AACTCGCCTACATGAAGCTGGAATCGCTAGTAATCGCGGATCAGCATGCCG
CGGTGAATACGTTCCCGGGCCTTGTACACACCGCCCGTCACACCACGAGA

GTTTGTAACACCCGAAGTCG

Blast comparison results in EZBioClound:

Select hits by database — All | Valid names only | Excel | FASTA | EzEditor2

Tasks	Hit taxon name	Hit strain name	Accession	Similarity	Variation ratio	Hit taxonomy	Completeness (%)
⇄ ○	Priestia aryabhattai	B8W22(T)	EF114313	100.00	0/1383	Bacteria;Firmicutes;Bacilli;Bacillales;Bacillaceae;Priestia	100.0
⇄ ○	Priestia megaterium	NBRC 15308(T)	JJMH01000057	99.86	2/1383	Bacteria;Firmicutes;Bacilli;Bacillales;Bacillaceae;Priestia	100.0
⇄ ○	Priestia flexa	NBRC 15715(T)	BCVD01000224	99.06	13/1383	Bacteria;Firmicutes;Bacilli;Bacillales;Bacillaceae;Priestia	100.0
⇄ ○	Bacillus pseudoflexus	RC1(T)	FN999944	98.91	15/1376	Bacteria;Firmicutes;Bacilli;Bacillales;Bacillaceae;Bacillus	98.8
⇄ ○	Priestia qingshengii	G19(T)	JX293295	98.26	24/1383	Bacteria;Firmicutes;Bacilli;Bacillales;Bacillaceae;Priestia	96.3
⇄ ○	Priestia paraflexa	RC2(T)	FN999943	97.95	28/1368	Bacteria;Firmicutes;Bacilli;Bacillales;Bacillaceae;Priestia	94.7

Blast comparison results in NCBI:

	Description	Scientific Name	Max Score	Total Score	Query Cover	E value	Per. Ident	Acc. Len	Accession
☑	Priestia aryabhattai strain kcgeb_S4 chromosome, complete genome	Priestia aryabhattai	2555	30616	100%	0.0	100.00%	5052464	CP145138.1
☑	Priestia sp. strain BAF 16S ribosomal RNA gene, partial sequence	Priestia sp.	2555	2555	100%	0.0	100.00%	1392	PP334154.1
☑	Priestia megaterium strain 39-2-4 16S ribosomal RNA gene, partial sequence	Priestia megaterium	2555	2555	100%	0.0	100.00%	1450	PP325800.1
☑	Priestia megaterium strain 21-1 16S ribosomal RNA gene, partial sequence	Priestia megaterium	2555	2555	100%	0.0	100.00%	1437	PP325797.1
☑	Priestia megaterium strain 13-2 16S ribosomal RNA gene, partial sequence	Priestia megaterium	2555	2555	100%	0.0	100.00%	1459	PP325794.1
☑	Priestia megaterium strain 13-1 16S ribosomal RNA gene, partial sequence	Priestia megaterium	2555	2555	100%	0.0	100.00%	1444	PP325793.1
☑	Priestia aryabhattai strain 178 16S ribosomal RNA gene, partial sequence	Priestia aryabhattai	2555	2555	100%	0.0	100.00%	1420	PP269442.1
☑	Bacillus sp. (in: firmicutes) strain 121 16S ribosomal RNA gene, partial sequence	Bacillus sp. (in: firmicutes)	2555	2555	100%	0.0	100.00%	1469	PP126481.1
☑	Bacillus sp. (in: firmicutes) strain PS02 16S ribosomal RNA gene, partial sequence	Bacillus sp. (in: firmicutes)	2555	2555	100%	0.0	100.00%	1470	PP126477.1
☑	Bacillus sp. (in: firmicutes) strain S07 16S ribosomal RNA gene, partial sequence	Bacillus sp. (in: firmicutes)	2555	2555	100%	0.0	100.00%	1466	PP126476.1
☑	Priestia sp. strain GR-1 16S ribosomal RNA gene, partial sequence	Priestia sp.	2555	2555	100%	0.0	100.00%	1461	PP112102.1
☑	Bacillus zanthoxyli strain SYS-Y53 16S ribosomal RNA gene, partial sequence	Bacillus zanthoxyli	2555	2555	100%	0.0	100.00%	1431	PP087852.1
☑	Bacillus sp. (in: firmicutes) strain SYS-Y49 16S ribosomal RNA gene, partial sequence	Bacillus sp. (in: firmicutes)	2555	2555	100%	0.0	100.00%	1444	PP087848.1
☑	Priestia megaterium strain HNS-S40 16S ribosomal RNA gene, partial sequence	Priestia megaterium	2555	2555	100%	0.0	100.00%	1454	PP087051.1
☑	Priestia aryabhattai strain cd28 16S ribosomal RNA gene, partial sequence	Priestia aryabhattai	2555	2555	100%	0.0	100.00%	1426	PP033750.1
☑	Priestia megaterium strain MS252 16S ribosomal RNA gene, partial sequence	Priestia megaterium	2555	2555	100%	0.0	100.00%	1467	PP023512.1

(2) Bacillus velezensis

16S rRNA sequencing:

GACAGATGGGAGCTTGCTCCCTGATGTTAGCGGCGGACGGGTGAGTAACACGTGGGTA
ACCTGCCTGTAAGACTGGGATAACTCCGGGAAACCGGGGCTAATACCGGATGGTTGTC
TGAACCGCATGGTTCAGACATAAAAGGTGGCTTCGGCTACCACTTACAGATGGACCCG
CGGCGCATTAGCTAGTTGGTGAGGTAACGGCTCACCAAGGCGACGATGCGTAGCCGAC
CTGAGAGGGTGATCGGCCACACTGGGACTGAGACACGGCCCAGACTCCTACGGGAGG
CAGCAGTAGGGAATCTTCCGCAATGGACGAAAGTCTGACGGAGCAACGCCGCGTGAG
TGATGAAGGTTTTCGGATCGTAAAGCTCTGTTGTTAGGGAAGAACAAGTGCCGTTCAA
ATAGGGCGGCACCTTGACGGTACCTAACCAGAAAGCCACGGCTAACTACGTGCCAGCA
GCCGCGGTAATACGTAGGTGGCAAGCGTTGTCCGGAATTATTGGGCGTAAAGGGCTCG
CAGGCGGTTTCTTAAGTCTGATGTGAAAGCCCCCGGCTCAACCGGGGAGGGTCATTGG
AAACTGGGGAACTTGAGTGCAGAAGAGGAGAGTGGAATTCCACGTGTAGCGGTGAAA
TGCGTAGAGATGTGGAGGAACACCAGTGGCGAAGGCGACTCTCTGGTCTGTAACTGA

CGCTGAGGAGCGAAAGCGTGGGGAGCGAACAGGATTAGATACCCTGGTAGTCCACGC

CGTAAACGATGAGTGCTAAGTGTTAGGGGGTTTCCGCCCCTTAGTGCTGCAGCTAACG

CATTAAGCACTCCGCCTGGGGAGTACGGTCGCAAGACTGAAACTCAAAGGAATTGAC

GGGGGCCCGCACAAGCGGTGGAGCATGTGGTTTAATTCGAAGCAACGCGAAGAACCT

TACCAGGTCTTGACATCCTCTGACAATCCTAGAGATAGGACGTCCCCTTCGGGGGCAG

AGTGACAGGTGGTGCATGGTTGTCGTCAGCTCGTGTCGTGAGATGTTGGGTTAAGTCC

CGCAACGAGCGCAACCCTTGATCTTAGTTGCCAGCATTCAGTTGGGCACTCTAAGGTG

ACTGCCGGTGACAAACCGGAGGAAGGTGGGGATGACGTCAAATCATCATGCCCCTTAT

GACCTGGGCTACACACGTGCTACAATGGACAGAACAAAGGGCAGCGAAACCGCGAGG

TTAAGCCAATCCCACAAATCTGTTCTCAGTTCGGATCGCAGTCTGCAACTCGACTGCGT

GAAGCTGGAATCGCTAGTAATCGCGGATCAGCATGCCGCGGTGAATACGTTCCCGGGC

CTTGTACACACCGCCCGTCACACCACGAGAGTTTGTA

Blast comparison results in EZBioClound:

Select hits by database — All | Valid names only | Excel | FASTA | EzEditor2

Tasks	Hit taxon name	Hit strain name	Accession	Similarity	Variation ratio	Hit taxonomy	Completeness (%)
	Bacillus siamensis	KCTC 13613(T)	AJVF01000043	99.85	2/1364	Bacteria;Firmicutes;Bacilli;Bacillales;Bacillaceae;Bacillus	100.0
	Bacillus velezensis	CR-502(T)	AY603658	99.85	2/1363	Bacteria;Firmicutes;Bacilli;Bacillales;Bacillaceae;Bacillus	95.4
	Bacillus subtilis	NCIB 3610(T)	ABQL01000001	99.71	4/1364	Bacteria;Firmicutes;Bacilli;Bacillales;Bacillaceae;Bacillus	100.0
	Bacillus amyloliquefaciens	DSM 7(T)	FN597644	99.63	5/1364	Bacteria;Firmicutes;Bacilli;Bacillales;Bacillaceae;Bacillus	100.0
	Bacillus nakamurai	NRRL B-41091(T)	LSAZ01000028	99.56	6/1364	Bacteria;Firmicutes;Bacilli;Bacillales;Bacillaceae;Bacillus	100.0
	Bacillus tequilensis	KCTC 13622(T)	AYTO01000043	99.49	7/1364	Bacteria;Firmicutes;Bacilli;Bacillales;Bacillaceae;Bacillus	100.0

Blast comparison results in NCBI:

Description	Scientific Name	Max Score	Total Score	Query Cover	E value	Per. Ident	Acc. Len	Accession
Priestia aryabhattai strain kcgeb_S4 chromosome, complete genome	Priestia aryabhattai	2555	30616	100%	0.0	100.00%	5052464	CP145138.1
Priestia sp. strain BAF 16S ribosomal RNA gene, partial sequence	Priestia sp.	2555	2555	100%	0.0	100.00%	1392	PP334154.1
Priestia megaterium strain 39-2-4 16S ribosomal RNA gene, partial sequence	Priestia megaterium	2555	2555	100%	0.0	100.00%	1450	PP325800.1
Priestia megaterium strain 21-1 16S ribosomal RNA gene, partial sequence	Priestia megaterium	2555	2555	100%	0.0	100.00%	1437	PP325797.1
Priestia megaterium strain 13-2 16S ribosomal RNA gene, partial sequence	Priestia megaterium	2555	2555	100%	0.0	100.00%	1459	PP325794.1
Priestia megaterium strain 13-1 16S ribosomal RNA gene, partial sequence	Priestia megaterium	2555	2555	100%	0.0	100.00%	1444	PP325793.1
Priestia aryabhattai strain 178 16S ribosomal RNA gene, partial sequence	Priestia aryabhattai	2555	2555	100%	0.0	100.00%	1420	PP269442.1
Bacillus sp. (in: firmicutes) strain 121 16S ribosomal RNA gene, partial sequence	Bacillus sp. (in: firmicutes)	2555	2555	100%	0.0	100.00%	1469	PP126481.1
Bacillus sp. (in: firmicutes) strain PS02 16S ribosomal RNA gene, partial sequence	Bacillus sp. (in: firmicutes)	2555	2555	100%	0.0	100.00%	1470	PP126477.1
Bacillus sp. (in: firmicutes) strain S07 16S ribosomal RNA gene, partial sequence	Bacillus sp. (in: firmicutes)	2555	2555	100%	0.0	100.00%	1466	PP126476.1
Priestia sp. strain GR-1 16S ribosomal RNA gene, partial sequence	Priestia sp.	2555	2555	100%	0.0	100.00%	1461	PP112102.1
Bacillus zanthoxyli strain SYS-Y53 16S ribosomal RNA gene, partial sequence	Bacillus zanthoxyli	2555	2555	100%	0.0	100.00%	1431	PP087852.1
Bacillus sp. (in: firmicutes) strain SYS-Y49 16S ribosomal RNA gene, partial sequence	Bacillus sp. (in: firmicutes)	2555	2555	100%	0.0	100.00%	1444	PP087848.1
Priestia megaterium strain HNS-S40 16S ribosomal RNA gene, partial sequence	Priestia megaterium	2555	2555	100%	0.0	100.00%	1454	PP087051.1
Priestia aryabhattai strain cd28 16S ribosomal RNA gene, partial sequence	Priestia aryabhattai	2555	2555	100%	0.0	100.00%	1426	PP033750.1
Priestia megaterium strain MS252 16S ribosomal RNA gene, partial sequence	Priestia megaterium	2555	2555	100%	0.0	100.00%	1467	PP023512.1

(3) Aspergillus fumigatus

ITS sequencing:

CCACCCGTGTCTATCGTACCTTGTTGCTTCGGCGGGCCCGCCGTTTCGACGGCCGCCGGGGAGGCCTT

GCGCCCCCGGGCCCGCGCCCGCCGAAGACCCCAACATGAACGCTGTTCTGAAAGTCTGCAGTCTGAG

TTGATTATCGTAATCAGTTAAAACTTTCAACAACGGATCTCTTGGTTCCGGCATCGATGAAGAACGCAG

CGAAATGCGATAAGTAATGTGAATTGCAGAATTCAGTGAATCATCGAGTCTTTGAACGCACATTGCGCC

CCCTGGTATTCCGGGGGGCATGCCTGTCCGAGCGTCATTGCTGCCCTCAAGCACGGCTTGTGTGTTGG

GCCCCCGTCCCCCTCTCCCGGGGGACGGGCCCGAAAGGCAGCGGCGGCACCGCGTCCGGTCCTCGAG

CGTATGGGGCTTTGTCACCTGCTCTGTAGGCCCGGCCGGCGCCAGCCGACACCCAACTTTATTTTTCTA

AGGTTGACCTCGGATCAGGTAGGGATACCCGCTGAACTTAA

Blast comparison results in NCBI:

Description	Name	Name	Taxid	Score	Score	Cover	value	Ident	Len	Accession
Aspergillus fumigatus clone SF_86 internal transcribed spacer 1, partial sequence; 5.8S ribosomal RNA ...	Aspergill...	NA	746128	957	957	100%	0.0	100.00%	563	MT529362.1
Aspergillus fumigatus strain 3162956_10__5_ITS5 internal transcribed spacer 1, partial sequence; 5.8S r...	Aspergill...	NA	746128	957	957	100%	0.0	100.00%	588	MH540723.1
Aspergillus fumigatus strain 3162953_F4_A_ITS5 internal transcribed spacer 1, partial sequence; 5.8S ri...	Aspergill...	NA	746128	957	957	100%	0.0	100.00%	578	MH540722.1
Aspergillus fumigatus strain 3162954_F3_A_ITS5 small subunit ribosomal RNA gene, partial sequence; i...	Aspergill...	NA	746128	957	957	100%	0.0	100.00%	587	MH540721.1
Aspergillus fumigatus strain CMXY28940 internal transcribed spacer 1, partial sequence; 5.8S ribosomal...	Aspergill...	NA	746128	957	957	100%	0.0	100.00%	586	MG991663.1
Aspergillus sp. strain Fi1 internal transcribed spacer 1, partial sequence; 5.8S ribosomal RNA gene and ...	Aspergill...	NA	5065	957	957	100%	0.0	100.00%	553	MG098706.1
Aspergillus fumigatus small subunit ribosomal RNA gene, partial sequence; internal transcribed spacer ...	Aspergill...	NA	746128	957	957	100%	0.0	100.00%	599	KX098468.1
Aspergillus sp. isolate CV00029 small subunit ribosomal RNA gene, partial sequence; internal transcrib...	Aspergill...	NA	5065	957	957	100%	0.0	100.00%	590	OR081395.1
Aspergillus fumigatus isolate 02436 internal transcribed spacer 1, partial sequence; 5.8S ribosomal RNA...	Aspergill...	NA	746128	957	957	100%	0.0	100.00%	563	OP630595.1
Aspergillus fumigatus strain AsfuHB24N01 internal transcribed spacer 1, partial sequence; 5.8S ribosom...	Aspergill...	NA	746128	957	957	100%	0.0	100.00%	565	OM372739.1
Aspergillus fumigatus strain ZH1 18S ribosomal RNA gene, partial sequence; internal transcribed spacer...	Aspergill...	NA	746128	957	957	100%	0.0	100.00%	597	JQ767180.1
Aspergillus fumigatus isolate NRRL 35223 18S ribosomal RNA gene, partial sequence; internal transcrib...	Aspergill...	NA	746128	957	957	100%	0.0	100.00%	1154	EF634403.1
Aspergillus fumigatus IBL 03108 internal transcribed spacer 1, partial sequence; 5.8S ribosomal RNA ge...	Aspergill...	NA	746128	957	957	100%	0.0	100.00%	571	DQ778906.1
Aspergillus fumigatus strain ZJ20C03 internal transcribed spacer 1, partial sequence; 5.8S ribosomal RN...	Aspergill...	NA	746128	952	952	100%	0.0	99.81%	582	PP385754.1
